# Comparison of Three Feeding Regimens on Blood Fatty Acids Metabolites of Wujumqin Sheep in Inner Mongolia

**DOI:** 10.3390/ani11041080

**Published:** 2021-04-10

**Authors:** Yanmei Jin, Xiaoqing Zhang, Jize Zhang, Qian Zhang, Tana

**Affiliations:** 1 Marine College, Shandong University, Weihai 264209, China; jinym2001@sohu.com; 2 Institute of Grassland Research, Chinese Academy of Agricultural Sciences, Hohhot 010010, China; zhangjize@caas.cn (J.Z.); zhangqian05@caas.cn (Q.Z.); tana@caas.cn (T.)

**Keywords:** feeding regimen, sheep, blood metabolite, fatty acids

## Abstract

**Simple Summary:**

The traditional sheep feeding system in Inner Mongolia, based on pasture grazing, is gradually transforming into a semi-grazing plus supplementation or feedlot approach, as grassland ecological protection becomes increasingly important. The fatty acid composition of the animals’ tissues changes with transformation of the feeding system. However, the changes to blood fatty acid metabolites in sheep as a result of alterations to the feeding regimen are unknown. In this study, pasture feeding, pasture feeding plus corn supplementation, and barn feeding were carried out to explore the effects of feeding regimens on blood fatty acid composition and metabolic pathways of sheep using a metabolomic approach. The results revealed that compared to grazing, concentrate supplement feeding regimens, including either grazing plus supplements or feeding indoors, down-regulated blood n-3 PUFA biosynthesis and up-regulated blood inflammatory compound metabolism by n-6 PUFA. These data suggest that under different feeding regimens, an appropriate ratio of n-6/n-3 PUFA in ruminant diets will contribute to increasingly high-quality animal production and improved immunocompetence.

**Abstract:**

Feeding regimens influence the fatty acid composition of animal-derived products. However, there is limited information on the effect of feeding regimens on the blood fatty acid composition and metabolic pathways of ruminant animals. In this study, 30 Wujumqin sheep were randomly assigned to three groups, PF (pasture feeding), PSF (pasture feeding plus corn supplementation) and BF (barn feeding), to examine the effects of feeding regimens on blood fatty acid composition and metabolic pathways through a metabolomic approach. The results showed that the BF sheep had increased serum n-6 polyunsaturated fatty acids levels, while the PF and PSF sheep had increased serum n-3 PUFA levels. Compared to the BF and PSF sheep that were fed ground corn, the PF sheep that only ate natural grass had up-regulated serum DHA levels. Meanwhile, blood metabolites from linoleic acid and arachidonic acid, including pro-inflammatory products (20-HETE, LTs, TX etc.) and anti-inflammatory products (LXB4, DHETs, HPETEs etc.) were elevated in the BF group. It was found that, compared to grazing, concentrate supplement feeding regimens, including either grazing plus supplements or feeding indoors, down-regulated blood n-3 PUFA biosynthesis and up-regulated the blood inflammatory compound metabolism by n-6 PUFA.

## 1. Introduction

A typical steppe region, Xilinguole League, accounting for 25% of the total grassland in Inner Mongolia with an area of 19.3 × 10^6^ ha, is considered as one of the most popular sheep production regions in China and even in the whole of the Eurasian grasslands [1]. However, because of drought stress caused by climate change and unreasonable animal husbandry, 74% of the grasslands in Xilinguole League have suffered from severe deterioration [2]. Simultaneously, the livestock population has grown dramatically, increasing by a factor of five over the past six decades [3], as a result of increases in meat and animal product demand and population pressure. Therefore, the need to develop sustainable grazing regimens for livestock has become urgent, to satisfy meat production needs while minimizing the environmental impacts.

The traditional sheep feeding system in Inner Mongolia is based on ad libitum grazing in pastures, which is gradually transforming into a semi-grazing plus supplementation or feedlot approach, as grassland ecological protection becomes more important, and with the implementation of the “Eco-green and High-quality Development Policy” by the central government of China. In practice, a great majority of herders used corn-based concentrates to feed animals, usually locally available at low cost for supplementation after grazing. Supplementation has a “substitution effect”, which can alleviate the pressure on grass growth in grasslands, reduce nutritional deficiencies from reliance on herbage, stimulate intake and digestibility and thus improve animals’ performance [4]. However, supplementation may alter the animals’ fatty acid (FA) metabolites. Studies have established that feeding regimens influence FA composition of animal-derived products [5,6]. For example, grazing sheep have been shown to contain less saturated fatty acids (SFA) and monounsaturated fatty acids (MUFA) but more polyunsaturated fatty acids (PUFA) in their meat, especially more long-chain n-3 PUFA (mainly eicosapentaenoic acid (EPA, C20:5n-3) and docosahexaenoic acid (DHA, C22:6n-3)) and a more favorable n-6/n-3 PUFA ratio [7,8] than sheep fed concentrate in a barn. Grazing increases the meat n-3 PUFA content because of the high proportion of α-linolenic acid (ALA, C18:3n-3) in natural grass [9], whereas indoor feeding increases the meat n-6 PUFA content due to the large amount of linoleic acid (LA, C18:2n-6) in corn-based concentrate [10]. An early study stated that the synthesis and metabolism of FA in the rumen and tissues of ruminant animals can be determined by the properties and metabolic pathways of the FA in the blood [11]. However, there is limited information on the effect of feeding regimens on the blood FA composition and metabolic pathways of ruminant animals.

The current study aimed to compare the effect of three feeding regimens—grazing, grazing plus corn supplementation and barn feeding—on the blood FA composition and the metabolic pathways (synthesis and metabolism) of n-6/n-3 PUFA of Chinese Wujumqin sheep. We hypothesized that corn supplementation or feeding indoors would down-regulate blood n-3 PUFA biosynthesis, and up-regulate blood n-6 PUFA, resulting in inflammatory compound metabolism in sheep.

## 2. Materials and Methods

### 2.1. Animals and Diets

The experiment was carried out in a research station located in Maodeng, which is typical grassland area in eastern Xilinguole (116°30′ E, 44°49′ N; alt. 1200 m a.s.l.), Inner Mongolia, China. The herbage of grassland in the region consists of *Stipa krylovii*, *Leymus chinensis*, *Cleistogenes squarrosa*, *Carex duriuscula* and *Allium ramosum*, which make up the usual botanical composition on offer in July–September and provide the bulk of the food for grazing sheep. The most abundant forage is in August, when the annual species *Salsola collina* and *Chenopodium album* are also present in scattered and patchy distributions. Herbage allowance of this pasture was 680 kg/ha in July, 927 kg/ha in August and 1040 kg/ha in September [12].

The experimental procedure was in accordance with the ethical standards of the Welfare and Ethics Committee of the Chinese Association for Laboratory Animal Sciences (Approval No. 20180330c1041215). In total, 30 Wujumqin adult female sheep, approximately 1.5 years of age, were obtained from a local farm. Prior to this experiment, all sheep grazed together on the same pasture. At the subsequent start of the experiment, sheep were randomly allocated into one of the three feeding groups (10 per group). The three feeding regime groups were: pasture feeding (PF, control), pasture feeding plus ground corn supplementation (PSF) and barn feeding (BF). From June to September, the PF group sheep were allowed to graze freely on pasture; the PSF group sheep grazed on the same pasture and were separately fed a supplement of 300 g of ground corn per head per day when off pasture, during the month of September. The BF sheep were fed indoors, with free access to *Chrysopogon aciculatus* hay and cornstalks and a daily feed of 500 g of ground corn per sheep. All animals in each group were reared for 120 days.

### 2.2. Blood Sample Preparation

Blood samples were collected on the last day of the experiment, before the morning feed. The samples were taken from the jugular vein of six randomly selected sheep in each group. For each sheep, 10 mL of blood was collected via a vacuum collection tube. All tubes were immediately placed on ice and the blood was allowed to naturally clot for one hour. Then, the serum was separated from the blood via centrifugation at 3000 rpm for 15 min at 4 °C. The serum samples were collected and aliquoted into 2 mL plastic vials, which were stored at –80 °C for subsequent determination of metabolites.

### 2.3. Metabolomics Analysis

The blood samples were thawed at 4 °C before analysis. Then, the non-targeted metabolomics analysis was carried out using an ultra-performance liquid chromatography (UPLC) system (Waters Corp., UK) according to the methods described in Dunn et al. [13].

The liquid chromatography–mass spectrometry (LC-MS) data acquired were analyzed in Progenesis QI v2.2 (Waters Corp., Milford, MA, USA) and the R package, metaX, as described in Wen et al. [14]. Metabolites were identified based on the Kyoto Encyclopedia of Genes and Genomes (KEGG) and the Human Metabolome Database (HMDB) also analyzed by metaX. Furthermore, the construction, interaction and pathway analyses of potential biomarkers were performed with software from the KEGG and HMDB databases.

### 2.4. Statistical Analysis

For serum metabolites, multivariate analyses, including Principal Com-ponent Analysis (PCA) and Partial Least Square Discriminant Analysis (PLS-DA) and the variable importance of projection (VIP) were performed to determine the metabolic differences between the treatment groups. In addition, a one-way ANOVA (analysis of variance) with Tukey’s honestly significant difference (Tukey’s HSD) test was performed on the metabolomics data, in order to assess which metabolites were primarily involved in each of the various groups. The threshold of significance was set at *p* < 0.05.

## 3. Results

### 3.1. Metabolite Profiling of the PF, PSF and BF Groups

**Figure 1:**
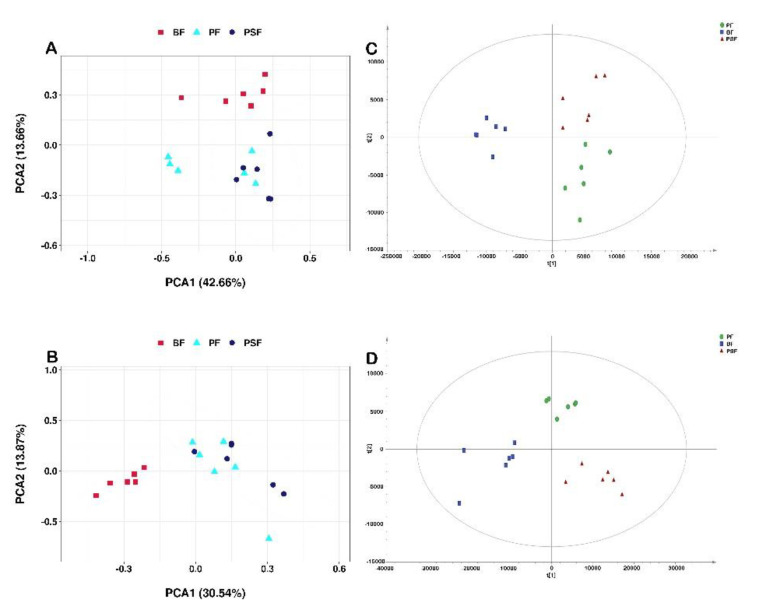
Multivariate data analyses of the liquid chromatography–mass spectrometry (LC-MS) serum spectra data. Principal Com-ponent Analysis (PCA) score plot analysis of the BF (barn feeding), PF (pasture feeding) and PSF (pasture feeding plus ground corn supplementation) groups (*n* = 6) in positive mode (**A**) and negative mode (**B**); Partial Least Square Discriminant Analysis (PLS-DA) S-plot in positive mode (**C**) and negative mode (**D**).

The PCA score plot showed that the serum metabolites in the BF group were clearly separated from the PF and PSF groups (Figure 1A,B). The PF group cluster was closer to that of the PSF group; however, differences were apparent. The supervised learning analysis, PLS-DA (Figure 1C,D), was subsequently performed to identify those metabolites contributing to the observed separation. In the PLS-DA score plot, the separation between the three groups was more prominent.

**Figure 2:**
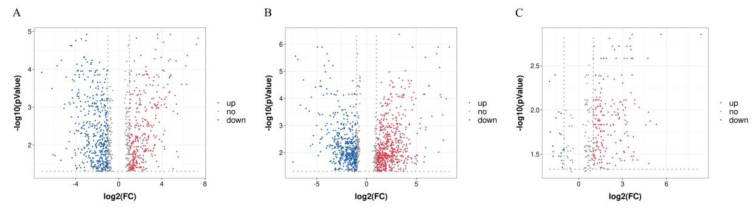
Volcano-plots of the metabolites in (**A**) the BF and the PF groups, (**B**) the PSF and BF groups and (**C**) the PSF and PF groups (*n* = 6).

Volcano plots were drawn for each pairwise comparison between groups. The *x*-axis is the mean ratio fold-change of the relative abundance of each metabolite, while the *y*-axis represents the statistical significance or *p*-value of the ratio fold-change for each metabolite. Points plotted in red had a fold-change ≥1.2 or ≤0.83 and *p* < 0.05, representing significantly different metabolites between the two groups. These results indicated that the different feeding regimens (i.e., BF vs. PF, PSF vs. BF, and PSF vs. PF) changed the serum metabolites of the sheep (Figure 2A–C).

### 3.2. Identification of Potential Metabolites and the Metabolic Pathways

Considering the VIP threshold (VIP ≥ 1) and *p*-value (*p* < 0.05), with a false discovery rate (FDR) < 0.05 and a fold change ≥ 1.2 or ≤ 0.83, a total of 909, 296 and 1218 significantly differentiated metabolites were identified among the three feeding regimes. Of these metabolites, 21, 23 and five, which potentially contributed the most to fatty acid metabolism, were selected for further investigation of BF vs. PF, BF vs. PSF, and PSF vs. PF, respectively.

**Figure 3:**
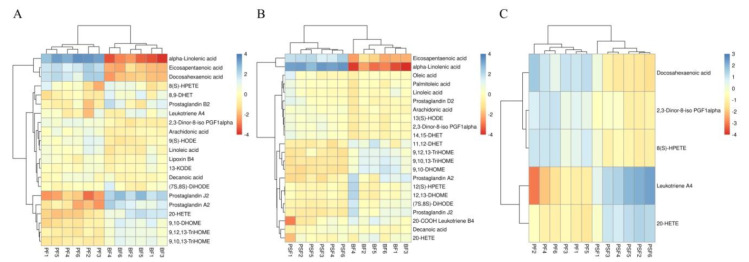
Heatmaps of the serum fatty acid (FA) potential biomarkers at the metabolite level of (**A**) the BF and the PF, (**B**) the PSF and BF and (**C**) the PSF and PF groups (*n* = 6). Each row represents a metabolite and each column represents a sheep sample.

Between the BF and PF groups, nine metabolites (Prostaglandin J2, 20-HETE, Prostaglandin A2, 8,9-DHET, Prostaglandin B2, Leukotriene A4, Lipoxin B4, 8(S)-HPETE and 2,3-Dinor-8-iso PGF1alpha) concerned with the arachidonic acid (AA, C20:4n-6) metabolism pathway and six metabolites (9,10-DHOME, 9,10-DHOME, 9,12,13-TriHOME, 13-KODE, (7S,8S)-DiHODE and 9(S)-HODE) involved in the LA metabolism pathway were significantly higher in the BF group. Metabolites involved in the biosynthesis of fatty acids, such as ALA, DHA and EPA, were significantly higher in the PF group, whereas decanoic acid (C10:0), LA, and AA were up-regulated in the BF group (Table 1, Figure 3A).

Of those 23 metabolites selected between the BF and PSF groups, nine compounds (11,12-DHET, prostaglandin J2, prostaglandin A2, 20-COOH-Leukotriene B4, 12(S)-HPETE, 20-HETE, 2,3-Dinor-8-iso PGF1alpha, 14,15-DHET, and thromboxane) related to the AA metabolism pathway and six compounds (9,10-DHOME, 9,10,13-TriHOME, 9,12,13-TriHOME, (7S,8S)-DiHODE, 12,13-DHOME, and 13(S)-HODE) related to the LA metabolism pathway were elevated in the BF group (Figure 3B). Most importantly, in comparison with the BF group, in the PSF group the SFAs, decanoic acid (C10:0) and stearic acid (C18:0), the MUFAs, palmitoleic acid (C16:1) and oleic acid (C18:1), as well as the n-6 PUFAs LA and AA, were down-regulated, while the n-3 PUFAs ALA and EPA were up-regulated (Table 2). 

The differential metabolites between the PSF and PF groups were mainly associated with AA metabolism. Among the four AA-related metabolites, leukotriene A4 and 20-HETE increased, while 2,3-Dinor-8-iso PGF1alpha and 8(S)-HPETE decreased in the PSF group. Interestingly, DHA, concerned with the biosynthesis of unsaturated fatty acids, was significantly up-regulated in the PF group (Table 3; Figure 3C). 

The up- and down-regulation of these metabolites and the related pathways are shown in Table 1, Table 2 and Table 3 and Figure 3.

In this table formula, retention time and m.z. of the differential metabolites are the same as in Table 1 and Table 2.

## 4. Discussion

### 4.1. Effect of Different Feeding Regimens on N-6 and N-3 PUFA Synthesis 

The metabolic pathway of unsaturated FA biosynthesis in sheep blood can be changed by feeding regimen. Indeed, in this study, BF resulted in an increased serum n-6 PUFA (mainly LA and AA) level, while both pasture-based feeding regimens (PF and PSF) increased the serum n-3 PUFA (mainly ALA and EPA) level of the sheep. Interestingly, the sheep in the PF group, which ate natural grass as their only dietary source, showed more elevated blood DHA levels than sheep in the PSF and BF groups, which were both fed ground corn.

Blood fatty acid levels largely depend on the dietary fatty acid sources, along with other factors like the ruminal bacterial synthesis and biohydrogenation (BH) of fatty acids, as well as endogenous conversion of ingested fatty acids in tissues by desaturation and elongation [11,15]. 

Diet plays an important role in the fat metabolism process. Characteristics of different dietary fatty acid types influence the bioavailability of fatty acids leaving the rumen and how easily they are absorbed in the small intestine for further incorporation into the body tissues of ruminants [16]. ALA, the most important dietary n-3 PUFA and the precursor for endogenous synthesis of EPA and DHA in animal tissues [10], is a major constituent of natural grass. LA, the most important dietary n-6 PUFA, is at a high level in corn-based concentrate feedstuff. Therefore, among the three groups in this study, the PF sheep had more ALA from natural grass in the pasture, while the BF sheep had more LA from their corn-based diet and the PSF sheep had moderate levels of both ALA and LA.

In the rumen, ALA and LA from the diet undergo BH and consequently are less readily absorbed in the intestine [15]. However, in grazing animals, fresh grass n-3 PUFAs may be more protected against rumen microorganisms than those in concentrates because of the presence of the secondary metabolites that could inhibit ruminal BH [17,18]. Consequently, a part of the dietary ALA in the PF and PSF groups may have passed through the rumen and been directly absorbed in the small intestine for further synthesis in the tissue.

PUFA synthesis in the tissue starts with Δ6 desaturation of both ALA and LA and both Δ5- and Δ6-desaturase play an important role in the extension of long-chain PUFA [19]. Fatty acids affect their own metabolism by affecting gene transcription. It has been reported that the enzymes Δ5- and Δ6-desaturase encoded by desaturase 1 (*FADS1*) and desaturase 2 (*FADS2*) genes were up-regulated in the tissues of grazing sheep due to the high proportion of ALA in natural grass [20,21]. In addition, the changes in mRNA expression of *FADS1* and *FADS2* were similar to those seen between EPA and DHA [20]. With more natural grass intake, there was less rumen BH activity and more active synthesis in the tissue, which led to the higher n-3 PUFA concentration, especially of ALA and EPA in the blood of the PF and PSF sheep, while more corn intake resulted in increased LA and AA concentrations in the blood of the BF sheep. Similar results were found by Popova et al. and Wang et al. [8,20], who reported that meat from pastured sheep contained more n-3 PUFA (ALA, EPA and DHA) compared to meat from sheep fed indoors. In theory, the PSF sheep should have had higher blood LA and AA concentrations than the PF sheep due to their greater n-6 PUFA intake from corn supplementation; however, this was not the case. This may have been due to the dose and short-time of supplementation (300 g/d only for a month), which may not have been sufficient to change the serum levels of LA and AA.

It is notable that the serum DHA level in the PF sheep was up-regulated more than that of the PSF sheep. The accumulation of DHA in the tissue is sensitive to the levels of both ALA and LA in the diet and can be regulated by altering the balance of n-6 and n-3 PUFA in the diet [22]. Ponnampalam et al. [23] and Gibson et al. [24] found that the synthesis and accumulation of DHA could be suppressed by LA because of competition between n-6 and n-3 PUFA at the enterocyte for absorption and metabolic enzymes. When grazing sheep consume corn supplements, their forage intake and digestion should be relatively lower [12]. Therefore, in this study, the PSF sheep consumed a much greater amount of LA and less ALA compared to the PF sheep who only consumed grass, which consequently led to a decreased DHA level in the blood of the PSF sheep. 

### 4.2. Effect of Different Feeding Regimens on PUFA Metabolism

Oxylipins, derived from n-6 PUFAs, such as LA and AA, form a complex pool of bioactive components [25], which serve as endogenous signaling molecules to regulate biological functions, including cell proliferation, inflammation and mediation of peripheral immune responses to systemic levels [26,27]. The hydroxy-octadecadienoic acids (HODEs), oxooctadecadienoic acids (KODEs) and trihydroxyoctadecenoic acids (TriHOMEs) are LA-derived end products for the LOX pathway, while dihydroxyoctadecenoic acids (DiHOMEs) and dihydroxyoctadecenoic acids (DHOMEs) [28,29] are LA-derived end-products for the CYP450 pathway. AA, converted from LA by Δ6-desaturation, can be metabolized into eicosanoids through different pathways and yields a plethora of compounds, such as prostaglandins (PG), isoprostanes, thromboxane (TX), leukotrienes (LT), lipoxins (LX), 8-12-15-hydroperoxyeicosatetraenoic acid (HPETE), epoxyeicosatrienoic acids (EETs), dihydroxyeicosatrienoic acids (DHETs) and 20-hydroxyeicosatetraenoic acid (20-HETE) [30]. Of these oxylipins, PGs, LTs, TXs, 20-HETE, 9-HODE, DiHODE and DHOMEs have pro-inflammatory actions, whereas LXs, PGJ eicosanoids, HPETE and 13-HODE are anti-inflammatory in nature [31,32]. The trihydroxyoctadecenoic acid, 9,10,13-TriHOME, is involved in regulating PGs synthesis. In this study, compared to the BF group, increased ALA intake in the PF and PSF sheep as a result of an increased amount of n-3 PUFA and a decreased amount of n-6 PUFA in the blood of those animals, led to a decrease in the production of inflammatory mediators, such as LA and AA-derived eicosanoids and cytokines. For example, the BF sheep demonstrated more active metabolism and up-regulated metabolites of LA and AA compared to the pastured sheep. Therefore, more blood metabolism compounds, such as 9-HODE, (7S,8S)-DiHODE and 9,10-DHOME, metabolized from LA and PGA2, as well as 2,3-Dinor-8-iso PGF1alpha, 20-HETE, LTs and TX, metabolized from AA, were produced by the BF sheep, which had pro-inflammatory effects on the animals. The anti-inflammatory products 13-HODE derived from LA and LXB4, DHETs, PGJ2 and HPETEs from AA were also elevated in the BF sheep. In comparison with the PF sheep, some pro-inflammatory products (i.e., 20-HETE and LT) were increased, but 2,3-Dinor-8-iso PGF1alpha was reduced, and the anti-inflammatory HPETEs metabolized from AA were down-regulated in the PSF group. Similar results were reported by Calder [33] and Hwang et al. [34], who found that when high levels of n-3 PUFA were consumed in the diet, less AA-derived eicosanoids were produced.

It is noteworthy that anti-inflammatory products inhibit the formation of pro-inflammatory products. In the present study, LA and AA gave rise to a range of mediators which have opposing effects to one another, suggesting that there likely exists a balance between pro-inflammatory and anti-inflammatory products formed from PUFAs. The overall physiological effect will be governed by the quantity of these mediators, the timing of their production and the sensitivities of target cells to their effects [35]. Thus, under normal physiological conditions, keeping a balance between the pro- and anti-inflammatory products seems to maintain homeostasis and prevent inappropriate inflammation [36]. 

The long-chain n-3 PUFAs, such as EPA and DHA, are recognized as functional lipids in anti-inflammatory immune responses [35]. A favorable ratio of n-3 PUFA (2.5 g/d) and n-6 PUFA (10 g/d) intake is also beneficial for human health [37] because the n-3 and n-6 PUFA groups compete as substrates for the same enzymes in the production of immuno-active eicosanoids [38]. Muturi et al. [39] reported that calves fed an n-3 PUFA-rich supplement or fed a diet with a low n-6/n-3 PUFA ratio can influence cellular mediators of immunity to nematode infection. For ruminants; however, n-3 and n-6 PUFA were commonly biohydrogenated by rumen microbes, resulting in inefficient conversion. How both excess n-6 and n-3 PUFA in the diet impacts immune responses remains unknown. Therefore, under different feeding regimens, as a component of control strategies, further studies are required to understand how manipulating the ratio of n-6/n-3 PUFA in ruminant diets contributes to increasingly high-quality animal production and the enhancement of immunocompetence.

## 5. Conclusions

The results from this blood metabolite study showed that, in comparison with grazing, concentrate supplementation to either grazing or indoor feeding regimes down-regulated blood n-3 PUFA biosynthesis and up-regulated blood inflammatory compound metabolism due to n-6 PUFA. Therefore, providing an appropriate ratio of dietary n-6/n-3 PUFA for ruminant animals will be beneficial for the synthesis and metabolism of fatty acids and to further improve the health of the animals.

## Figures and Tables

**Table 1:** Identification and trends of change for the differential metabolites in the BF and PF groups (*n* = 6).

Chemical Denomination	Formula	*p*-Value	VIP (Variable Importance of Projection)	m.z (Mass-to-Charge Ratio)	Retention Time (min)	Trend	Metabolic Pathway
2,3-Dinor-8-iso PGF1alpha	C_18_H_32_O_5_	2.18 × 10^−2^	1.06	327.22	7.57	up	Arachidonic acid metabolism
Leukotriene A4	C_20_H_30_O_3_	1.48 × 10^−2^	1.86	317.21	8.04	up	Arachidonic acid metabolism
Prostaglandin A2	C_20_H_30_O_4_	4.09 × 10^−4^	2.96	333.21	7.11	up	Arachidonic acid metabolism
Prostaglandin B2	C_20_H_30_O_4_	5.84 × 10^−3^	2.37	333.21	7.99	up	Arachidonic acid metabolism
Prostaglandin J2	C_20_H_30_O_4_	4.32 × 10^−5^	3.68	333.21	7.67	up	Arachidonic acid metabolism
20-HETE	C_20_H_32_O_3_	1.21 × 10^−5^	3.25	319.23	8.31	up	Arachidonic acid metabolism
8(S)-HPETE	C_20_H_32_O_4_	3.75 × 10^−2^	1.54	335.22	6.84	up	Arachidonic acid metabolism
Lipoxin B4	C_20_H_32_O_5_	8.00 × 10^−3^	1.56	351.22	7.25	up	Arachidonic acid metabolism
8,9-DHET	C_20_H_34_O_4_	1.61× 10^−4^	2.68	337.24	6.66	up	Arachidonic acid metabolism
13-KODE	C_18_H_30_O_3_	8.65 × 10^−4^	1.95	293.21	8.74	up	Linoleic acid metabolism
9(S)-HODE	C_18_H_32_O_3_	2.52 × 10^−2^	1.09	295.23	8.15	up	Linoleic acid metabolism
(7S,8S)-DiHODE	C_18_H_32_O_4_	1.05 × 10^−2^	1.93	311.22	8.23	up	Linoleic acid metabolism
9,10-DHOME	C_18_H_34_O_4_	4.88× 10^−5^	2.83	313.24	8.11	up	Linoleic acid metabolism
9,10,13-TriHOME	C_18_H_34_O_5_	1.51 × 10^−5^	2.70	329.23	7.25	up	Linoleic acid metabolism
9,12,13-TriHOME	C_18_H_34_O_5_	3.01 × 10^−5^	2.59	329.23	6.91	up	Linoleic acid metabolism
alpha-Linolenic acid	C_18_H_30_O_2_	2.38 × 10^−5^	3.08	277.22	8.44	down	Biosynthesis of unsaturated fatty acids
Linoleic acid	C_18_H_32_O_2_	4.08 × 10^−2^	1.13	279.23	9.33	up	Biosynthesis of unsaturated fatty acids
Eicosapentaenoic acid	C_20_H_30_O_2_	8.05 × 10^−4^	1.65	301.22	9.01	down	Biosynthesis of unsaturated fatty acids
Arachidonic acid	C_20_H_32_O_2_	5.3 9× 10^−3^	1.41	303.96	10.1	up	Biosynthesis of unsaturated fatty acids
Docosahexaenoic acid	C_22_H_32_O_2_	5.69 × 10^−3^	1.81	327.23	9.23	down	Biosynthesis of unsaturated fatty acids
Decanoic acid	C_10_H_20_O_2_	4.92 × 10^−4^	1.82	171.14	7.63	up	Fatty acid biosynthesis

**Table 2:** Identification and trends of change for the differential metabolites in the BF and PSF groups (*n* = 6).

Chemical Denomination	Formula	*p*-Value	VIP	m.z	Retention Time (min)	Trend	Metabolic Pathway
2,3-Dinor-8-iso PGF1alpha	C_18_H_32_O_5_	6.70 × 10^−^^3^	1.15	327.22	7.57	up	Arachidonic acid metabolism
Thromboxane	C_20_H_32_O_5_	1.91 × 10^−^^2^	1.12	387.20	9.16	up	Arachidonic acid metabolism
20-COOH-Leukotriene B4	C_20_H_30_O_6_	6.11 × 10^−^^3^	1.93	401.18	7.48	up	Arachidonic acid metabolism
14,15-DHET	C_20_H_34_O_4_	9.04 × 10^−^^3^	1.12	337.24	9.78	up	Arachidonic acid metabolism
11,12-DHET	C_20_H_34_O_4_	3.98 × 10^−^^4^	2.25	337.24	6.66	up	Arachidonic acid metabolism
12(S)-HPETE	C_20_H_32_O_4_	2.26 × 10^−^^2^	1.79	335.22	8.47	up	Arachidonic acid metabolism
Prostaglandin A2	C_20_H_30_O_4_	5.25 × 10^−^^3^	2.16	333.21	7.99	up	Arachidonic acid metabolism
Prostaglandin J2	C_20_H_30_O_4_	2.92 × 10^−^^3^	2.24	333.20	8.23	up	Arachidonic acid metabolism
20-HETE	C_20_H_32_O_3_	4.70 × 10^−^^3^	1.36	319.23	8.31	up	Arachidonic acid metabolism
13(S)-HODE	C_18_H_32_O_3_	7.38 × 10^−^^3^	1.24	295.23	8.15	up	Linoleic acid metabolism
(7S,8S)-DiHODE	C_18_H_32_O_4_	5.82 × 10^−^^3^	2.03	311.22	8.23	up	Linoleic acid metabolism
9,10-DHOME	C_18_H_34_O_4_	4.42 × 10^−^^5^	2.55	313.24	8.11	up	Linoleic acid metabolism
12,13-DHOME	C_18_H_34_O_4_	2.81 × 10^−^^2^	1.74	313.24	8.48	up	Linoleic acid metabolism
9,10,13-TriHOME	C_18_H_34_O_5_	1.77 × 10^−^^5^	2.42	329.23	7.25	up	Linoleic acid metabolism
9,12,13-TriHOME	C_18_H_34_O_5_	3.37 × 10^−^^4^	2.16	329.23	6.91	up	Linoleic acid metabolism
Linoleic acid	C_18_H_32_O_2_	1.04 × 10^−^^2^	1.40	279.23	9.33	up	Linoleic acid metabolism Biosynthesis of unsaturated fatty acids
alpha-Linolenic acid	C_18_H_30_O_2_	6.04 × 10^−^^5^	2.99	277.22	8.44	down	Biosynthesis of unsaturated fatty acids
Oleic acid	C_18_H_34_O_2_	1.84 × 10^−^^2^	1.29	281.25	9.66	up	Biosynthesis of unsaturated fatty acids
Stearic acid	C_18_H_36_O_2_	3.31 × 10^−^^2^	1.41	285.28	9.68	up	Biosynthesis of unsaturated fatty acids
Eicosapentaenoic acid	C_20_H_30_O_2_	3.65 × 10^−^^4^	1.82	301.22	9.01	down	Biosynthesis of unsaturated fatty acids
Arachidonic acid	C_20_H_32_O_2_	8.40 × 10^−^^4^	1.19	303.96	9.58	up	Biosynthesis of unsaturated fatty acids
Palmitoleic acid	C_16_H_30_O_2_	5.47 × 10^−^^3^	1.36	253.22	9.17	up	Fatty acid biosynthesis
Decanoic acid	C_10_H_20_O_2_	8.47 × 10^−^^4^	1.57	171.14	7.63	up	Fatty acid biosynthesis

**Table 3:** Identification and trends of change for the differential metabolites in the PSF and PF groups (*n* = 6).

Chemical Denomination	*p*-Value	VIP	Trend	Metabolic Pathway
2,3-Dinor-8-iso PGF1alpha	3.72 × 10^−^^2^	1.45	down	Arachidonic acid metabolism
Leukotriene A4	8.51 × 10^−^^3^	4.82	up	Arachidonic acid metabolism
20-HETE	1.09 × 10^−^^2^	2.93	up	Arachidonic acid metabolism
8(S)-HPETE	3.35 × 10^−^^2^	1.19	down	Arachidonic acid metabolism
Docosahexaenoic acid	3.32 × 10^−^^2^	2.47	down	Biosynthesis of unsaturated fatty acids
